# Supplementary material for: Genetic Variation and Composition of Two Commercial Estonian Dairy Cattle Breeds Assessed by SNP Data
Source: Animals (Basel). 2024 Apr 4;14(7):1101. doi: 10.3390/ani14071101 (PMC11010984; doi:10.3390/ani14071101)
Supplement: Supplementary file 1 [file animals-14-01101-s001.zip › animals-2906757-supplementary.pdf]

Supplementary information

Table S1. Genomic inbreeding coefficients (F_{HOM} and F_{ROH}) by sire-origin groups in Estonian Holstein and in Estonian Red breed.

Sire origin *	Group size	F_{HOM}		F_{ROH}	
		**Mean**	**Sd**	**Mean**	**Sd**
Estonian Holstein					
CAN	126	-0.0059	0.0302	0.1172	0.0263
CHE	33	-0.0167	0.0368	0.1040	0.0333
DEU	566	-0.0092	0.0291	0.1132	0.0263
DSF	260	-0.0082	0.0317	0.114	0.0276
inc. DNK	244	-0.0101	0.0308	0.1123	0.0266
FIN	11	0.0188	0.0367	0.1393	0.0281
SWE	5	0.0234	0.0263	0.1455	0.0343
EST	99	-0.0169	0.0247	0.1046	0.0210
FRA	21	-0.0132	0.0247	0.1168	0.0231
GBR	15	-0.0210	0.0164	0.1011	0.0113
NLD	488	-0.0105	0.0300	0.1110	0.0265
USA	653	0.0029	0.0298	0.1235	0.0265
**Total**	**2265**	**-0.0063**	**0.030**	**0.1155**	**0.0269**
Estonian Red					
DNK	89	-0.0135	0.0288	0.0428	0.0241
EST	32	-0.0173	0.0271	0.0432	0.0232
FIN	77	-0.0112	0.0345	0.0438	0.0289
SWE	18	-0.0050	0.0189	0.0474	0.0162
**Total**	**215**	**-0.013**	**0.0303**	**0.0438**	**0.0252**

*CAN – Canada, CHE – Switzerland, DEU – Germany, DFS – Denmark, Finland, Sweden, DNK – Denmark, EST – Estonia, FIN – Finland, FRA – France, GBR – Great Britain, NLD – Netherlands, SWE – Sweden, USA – United States of America.
